# Contemporary Analysis of Electronic Frailty Measurement in Older Adults with Multiple Myeloma Treated in the National US Veterans Affairs Healthcare System

**DOI:** 10.3390/cancers13123053

**Published:** 2021-06-18

**Authors:** Clark DuMontier, Nathanael R. Fillmore, Cenk Yildirim, David Cheng, Jennifer La, Ariela R. Orkaby, Brian Charest, Diana Cirstea, Sarvari Yellapragada, John Michael Gaziano, Nhan Do, Mary T. Brophy, Dae H. Kim, Nikhil C. Munshi, Jane A. Driver

**Affiliations:** 1 New England Geriatrics Research, Education and Clinical Center, VA Boston Healthcare System, Boston, MA 02130, USA; clark.dumontier2@va.gov (C.D.); ariela.orkaby@va.gov (A.R.O.); 2 Division of Aging, Brigham and Women’s Hospital, Boston, MA 02115, USA; michael.gaziano@va.gov; 3 Harvard Medical School, Boston, MA 02115, USA; nathanael.fillmore@va.gov (N.R.F.); nikhil_munshi@dfci.harvard.edu (N.C.M.); 4 VA Boston CSP Center, Boston, MA 02130, USA; nhan.do@va.gov (N.D.); mary.brophy@va.gov (M.T.B.); 5 Massachusetts Veterans Epidemiology Research and Information Center (MAVERIC), Boston, MA 02130, USA; cenk.yildirim@va.gov (C.Y.); jennifer.la3@va.gov (J.L.); brian.charest@va.gov (B.C.); 6 VA Boston Healthcare System, Boston, MA 02130, USA; 7 Department of Medical Oncology, Dana-Farber Cancer Institute, Boston, MA 02215, USA; diana_cirstea@dfci.harvard.edu; 8 Massachusetts General Hospital, Boston, MA 02114, USA; dcheng@mgh.harvard.edu; 9 Michael E. Debakey VA Medical Center and Dan L. Duncan Cancer Center, Baylor College of Medicine, Houston, TX 77030, USA; yellapra@bcm.edu; 10 Boston University School of Medicine, Boston, MA 02118, USA; 11 Marcus Institute for Aging Research, Hebrew SeniorLife, Boston, MA 02131, USA; daehyunkim@hsl.harvard.edu; 12 Division of Gerontology, Beth Israel Deaconess Medical Center, Boston, MA 02215, USA

**Keywords:** frailty, frailty index, multiple myeloma, geriatric assessment

## Abstract

**Simple Summary:**

Geriatric and frailty assessment are recommended for all older adults with cancer undergoing systemic therapy, but assessments remain difficult to scale. The aim of this study was to use an electronic frailty index based on data from administrative claims and electronic health records—the Veterans Affairs Frailty Index (VA-FI-10)—to estimate frailty and its impact on older United States (US) military veterans treated for multiple myeloma (MM) throughout the national VA Healthcare System. We found frailty to be prevalent and strongly associated with mortality and hospitalizations—independently of age, race, and MM stage. We also showed that changing the way in which the VA-FI-10 is measured affects its classification of frailty for individual veterans but not its association with mortality. These findings support the VA-FI-10’s use in research investigating outcomes in frail veterans treated with contemporary MM therapies. We provide further insights into the VA-FI-10’s potential use in clinical practice.

**Abstract:**

Electronic frailty indices based on data from administrative claims and electronic health records can be used to estimate frailty in large populations of older adults with cancer where direct frailty measures are lacking. The objective of this study was to use the electronic Veterans Affairs Frailty Index (VA-FI-10)—developed and validated to measure frailty in the national United States (US) VA Healthcare System—to estimate the prevalence and impact of frailty in older US veterans newly treated for multiple myeloma (MM) with contemporary therapies. We designed a retrospective cohort study of 4924 transplant-ineligible veterans aged ≥ 65 years initiating MM therapy within VA from 2004 to 2017. Initial MM therapy was measured using inpatient and outpatient treatment codes from pharmacy data in the VA Corporate Data Warehouse. In total, 3477 veterans (70.6%) were classified as frail (VA-FI-10 > 0.2), with 1510 (30.7%) mildly frail (VA-FI-10 > 0.2–0.3), 1105 (22.4%) moderately frail (VA-FI-10 > 0.3–0.4), and 862 (17.5%) severely frail (VA-FI-10 > 0.4). Survival and time to hospitalization decreased with increasing VA-FI-10 severity (log-rank *p*-value < 0.001); the VA-FI-10 predicted mortality and hospitalizations independently of age, sociodemographic variables, and measures of disease risk. Varying data sources and assessment periods reclassified frailty severity for a substantial portion of veterans but did not substantially affect VA-FI-10’s association with mortality. Our study supports use of the VA-FI-10 in future research involving older veterans with MM and provides insights into its potential use in identifying frailty in clinical practice.

## 1. Introduction

Geriatric assessment is now recommended by leading cancer organizations for all older adults with cancer undergoing systemic therapy [1,2,3]. For older adults with multiple myeloma (MM), assessment of frailty and function is essential in determining the intensity of the initial and subsequent MM regimens, which now range from two to four drugs for the majority of older patients who are ineligible for hematopoietic stem-cell transplant [4,5]. Older patients with MM identified as frail have a higher risk of treatment toxicity, higher rates of treatment discontinuation, and higher mortality [6,7,8,9]. Diagnosing frailty not only aids in MM treatment selection, but also identifies aging-related deficits that can be addressed and optimized alongside MM treatment [6,10]. Despite its benefits, widespread implementation of geriatric and frailty assessment in oncology research and practice remains limited by a lack of time, staff, and other resources [11]. Without rigorous and widespread frailty assessment, little is known about the outcomes in frail older patients who are treated with MM regimens that have predominantly been studied in younger and fitter trial populations [12].

Electronic frailty indices (eFIs) have been proposed as one strategy to overcome the lack of frailty measurement in clinical practice and research databases [13,14,15,16]. Instead of in-person frailty assessment—requiring a broad array of patient-reported and/or objective performance measures of morbidity, cognition, and function—an eFI can estimate frailty using data readily available from administrative claims and electronic health records (EHRs) [17]. As an example, the United Kingdom’s National Health Service (NHS) calculates an eFI to screen for frailty in all older patients seen throughout its nationalized healthcare system [18]. In the United States (US), the Veterans Affairs (VA) Healthcare System is the nation’s largest integrated health system, caring for over 9 million veterans each year in over 1200 facilities [19]. The electronic VA Frailty Index (VA-FI) was developed in millions of older US Veterans for the purpose of measuring frailty using data from claims and EHR [20]. In 2021, the VA-FI was updated to incorporate ICD-10 codes (VA-FI-10) for contemporary measurement of frailty in veterans [21].

The primary aim of this study was to use the VA-FI-10 to estimate the prevalence and impact of frailty on mortality and hospitalizations in transplant-ineligible older US veterans with MM newly treated with contemporary therapies throughout VA. In contrast to more universal healthcare systems, such as the NHS, the US healthcare system provides a substantial portion of its healthcare services through the private sector; veterans can access services internally through the national VA or externally through non-VA facilities [22]. It is unclear how this external healthcare utilization affects the VA-FI-10’s measurement in US veterans with MM and other cancers. Moreover, although other studies have shown that varying the length of assessment periods in which to measure comorbid conditions may change their prevalence and association with outcomes [23,24], it is unclear how varying assessment periods affects the VA-FI-10 in veterans with MM. Accordingly, the secondary aim of this study was to assess how the VA-FI-10 is affected by varying data sources and assessment periods in which health deficits are captured both within and outside VA. We hypothesized that the VA-FI-10 would predict mortality and hospitalizations, and that external data and varying assessment periods would affect both the distribution of frailty and its associations with these outcomes.

## 2. Materials and Methods

### 2.1. Data Source and Population

We designed a retrospective cohort study analyzing data from the VA Corporate Data Warehouse (CDW), which collects clinical, billing, and EHR information from veterans treated in VA facilities throughout the United States [25]. To capture the health deficits managed outside VA, we linked the VA CDW with data from the Centers for Medicare and Medicaid Services (CMS) [26], which is the main healthcare payer for non-veteran adults aged ≥65 years. Further details regarding each database in terms of their measurement of data, their breadth of data, and their strengths and limitations can be found in the following references [27,28]. We selected veterans aged ≥65 years newly treated for MM throughout VA. Treatments included any medical therapy (any class of medical therapy for MM, including proteasome inhibitors, immune-modifying drugs, and chemotherapy). Patients who received a hematopoietic stem-cell transplant were excluded.

Consistent with our previous work, our inclusion criteria included at least three dates on which MM diagnostic codes were observed (International Classification of Diseases (ICD)-9: codes with prefix 203.0; ICD-10: codes with prefix C90.0) and at least two dates on which MM treatment was observed, with the second date falling within six months of the first [29]. This second treatment code better ensures that veterans are both beginning and continuing their treatment within VA versus receiving longitudinal treatment elsewhere in an external healthcare system. The index date on which the study follow-up started was the date of the first treatment code, representing the initiation of treatment. We limited our study to veterans with an index date falling within the years 2004 through 2017, matching the years of available CMS data at the time of analysis. We included veterans aged ≥65 years at the index date. To further limit our population to veterans who were more consistently utilizing VA, we required at least one VA non-MM diagnosis or procedure code in each year during the 3 years preceding the index date. To exclude veterans who had received prior MM treatment or who were being treated for their MM outside VA, we excluded veterans with one or more MM treatment codes in the CMS data prior to or up to six months after the index date (see Appendix A for further details).

### 2.2. Measurement of Frailty and Covariates

To measure frailty, we used the VA-FI-10: an eFI developed and validated in millions of US veterans ≥ 65 years old [20,21]. In brief, the VA-FI-10 measures frailty based on the deficit-accumulation approach—one of the most widely studied models of frailty [30]. In total, 31 aging-related health deficits are measured from administrative claims and EHR data, using diagnostic and procedural codes spanning the domains of morbidity (14 deficits), function [8], cognition [3], sensory [3], and other [3,21] (see Appendix A for further detail on number and types of codes). These health deficits are measured within a 3-year assessment period leading up to the date of treatment initiation; deficits captured within VA (internally) or CMS (external to VA) are only counted once. A score is calculated for each patient as the proportion of all health deficits present over the total deficits possible, ranging from 0–1 where higher values indicate more severe frailty [31]. Based on validated cut-points [20,32,33,34,35], the VA-FI-10 categories were non-frail (VA-FI-10 ≤ 0.1), pre-frail (VA-FI-10 > 0.1 to 0.2), mildly frail (VA-FI-10 > 0.2 to 0.3), moderately frail (VA-FI-10 > 0.3 to 0.4), and severely frail (VA-FI-10 > 0.4).

Covariates were extracted from the VA CDW and included the sociodemographic variables age at initiation of treatment, sex, race, and income. Measures of disease risk included laboratory data related to MM stage and prognosis, measured in a time period starting 90 days before the index date with the latest value being used. MM stage was classified using the MM International Staging System based on serum albumin and beta-2 microglobulin [36]. Calcium, creatinine, hemoglobin, and platelet levels were also measured using prespecified cutoffs validated in the literature [37]. MM therapies were measured using inpatient and outpatient treatment codes from pharmacy data in the VA Corporate Data Warehouse. Specific therapies were identified at time of treatment initiation, defined as the first 90 days after the index date. Among these therapies, we classified novel therapy as any proteasome inhibitor (bortezomib, carfilzomib, or ixazomib) or immunomodulatory agent (thalidomide, lenalidomide, or pomalidomide). We did not report dexamethasone or other steroids utilization since almost all patients received them as part of their induction regimens, given their inclusion in most treatment guidelines relevant to the time period of our study [38].

### 2.3. Outcomes

The primary outcome was overall survival, measured using vital status information in the VA CDW (which provides 98.3% sensitivity and 97.6% exact agreement against the US National Death Index) [39]. Our secondary outcome was unplanned hospitalizations (admissions not prescheduled, e.g., for elective surgery) within the VA Healthcare System, measured from the CDW. Veterans were followed through 30 June 2019 until death or their last record in the CDW, after which they were censored.

### 2.4. Statistical Analysis

Population characteristics were summarized using mean and standard deviation (SD), median and interquartile range, or proportions. We used Kaplan–Meier analyses and log-rank tests to assess whether the time to death and hospitalization differed across frailty categories. To assess for an independent association between VA-FI-10 and outcomes, we fit Cox proportional hazards regression models to estimate the hazard ratios (HRs) for mortality and time to hospitalization, adjusting for age, sociodemographic factors (gender, race, income, year of treatment initiation), and measures of disease risk (ISS stage and all baseline laboratory covariates defined above). These covariates were selected based on clinical knowledge and a priori evidence of prognostic factors in MM [37]. Year of treatment initiation was used as a surrogate to adjust for treatment evolution over time. To impute any missing baseline covariates from the available baseline data, we used multiple imputation with chained equations as implemented in the R MICE package [40,41]. We performed a complete case analysis as a sensitivity analysis. As an additional sensitivity analysis, we repeated our multivariable analyses in 2012–2017, the later time period of our study during which a higher proportion of patients were receiving modern-era treatment regimens.

We conducted secondary analyses to assess how the distribution and associations of the VA-FI-10 were affected by adding CMS data to VA CDW data and by using a 1-year assessment period instead of a 3-year assessment period. Paired t-tests were used to evaluate the mean intra-individual changes in VA-FI-10 between these variations. All statistical tests were 2-sided, and a *p*-value less than 0.05 was considered statistically significant. All analyses were performed using R 3.6.0 (R Foundation for Statistical Computing, Vienna, Austria). In reporting this study, we followed the guidelines put forth by the Strengthening the Reporting of Observational Studies in Epidemiology Statement [42].

## 3. Results

### 3.1. Baseline Characteristics of Study Population

**Figure 1:**
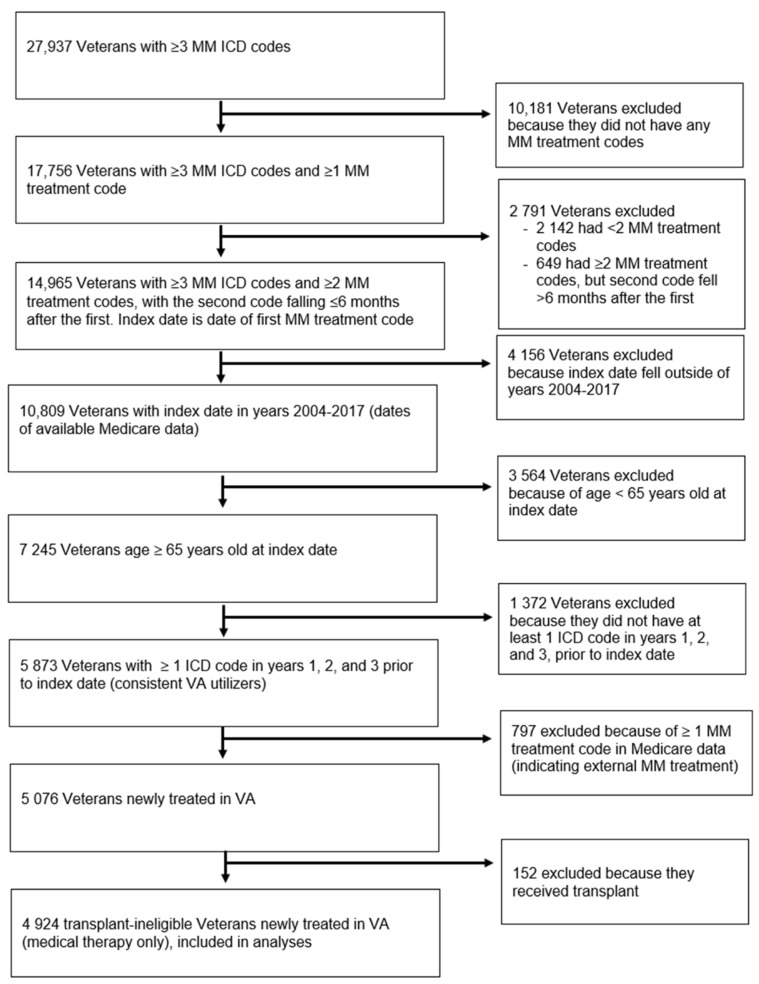
Flow diagram showing identification of the study population of veterans aged ≥65 years old with multiple myeloma newly treated in Veterans Affairs. Abbreviations: ICD, International Classification of Diseases; MM, multiple myeloma; VA, Veterans Affairs.

**Figure 2:**
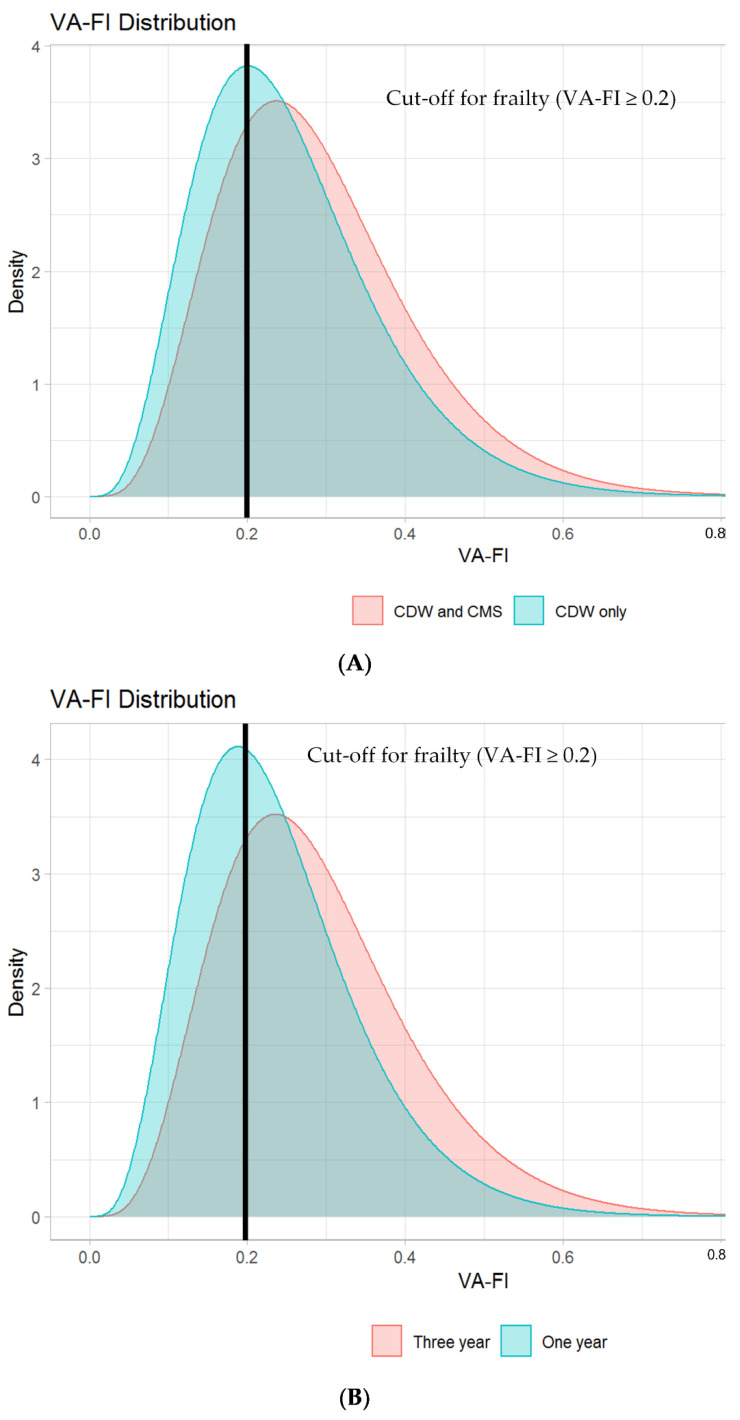
(**A**) Distribution of VA-FI-10 in 4924 transplant-ineligible older veterans treated with medical myeloma therapies, measured using data from both the VA Corporate Data Warehouse (CDW) and Centers for Medicare and Medicaid Services (CMS, red histogram), compared to the distribution of VA-FI-10 measured using data from the VA Corporate Data Warehouse only (blue histogram). (**B**) Distribution of VA-FI-10 measured using CDW and CMS data and a 3-year assessment period (red histogram) compared with the distribution of VA-FI-10 measured using CDW and CMS data and a 1-year assessment period (blue histogram).

Figure 1 displays the selection of our study population within the VA CDW, resulting in 4924 transplant-ineligible older veterans treated with medical MM therapies. The median time between the first and second MM treatment codes was 11 days (interquartile range (IQR) = 4–29 days). Table 1 displays the baseline characteristics by VA-FI-10 severity. Overall, 3477 veterans (70.6%) were classified as frail (VA-FI-10 > 0.2), with 1510 (30.7%) mildly frail (VA-FI-10 > 0.2 to 0.3), 1105 (22.4%) moderately frail (VA-FI-10 > 0.3 to 0.4), and 862 (17.5%) severely frail (VA-FI-10 > 0.4; Figure 2A, red histogram). The majority of veterans were male (98.6%). Median age increased across VA-FI-10 severity (median age 72.6 in non-frail veterans to 77.4 in severely frail veterans). Compared to non-frail patients, frail patients tended to have a lower median income and higher rates of lab abnormalities. The majority of veterans were treated with at least one novel therapy at induction (85.9%), with non-frail patients receiving the highest rates of novel therapy (90.4%).

Table 2 displays the baseline prevalence of VA-FI-10 health deficits by VA-FI-10 severity; Table S1 displays deficits by cohort year. In the morbidity domain, veterans had high rates of hypertension (prevalence range 86.3% to 89.0% from 2012–2017), anemia (66.5% to 71.3%), diabetes (40.5% to 46.9%), and chronic kidney disease (40.5% to 46.6%). In the functional domain, the majority of veterans had arthritis (51.3% to 60.6%), and a large proportion had muscular impairment (16.8% to 25.1%), gait abnormalities (20.4% to 25.3%), and a requirement for durable medical equipment (20.8% to 35.1%); the prevalence of each of these functional deficits rose to nearly 50% for severely frail veterans. In the cognitive domain, depression rates ranged from 24.5% to 31.7%, and dementia rates ranged from 11.2% to 17.0%; the prevalence of depression and dementia rose to 47.6% and 34.7%, respectively, in severely frail veterans. In the sensory domain, visual (27.3% to 33.8%) and hearing impairments (33.5% to 42.7%) were prevalent. About one-in-four to one-in-three veterans had chronic pain (24.6% to 33.4%).

### 3.2. Associations of VA-FI-10 with Mortality and Unplanned Hospitalizations

**Figure 3:**
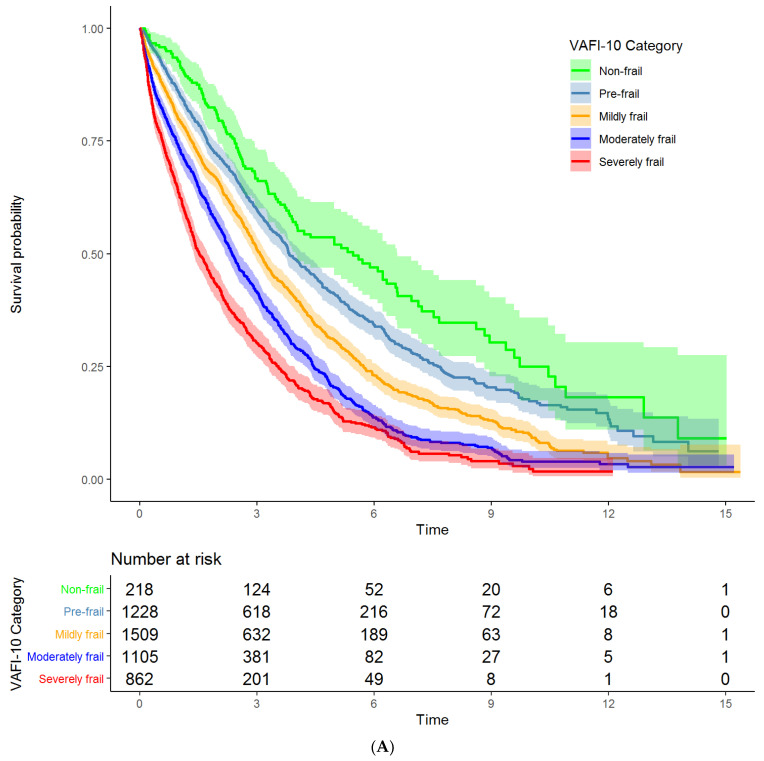

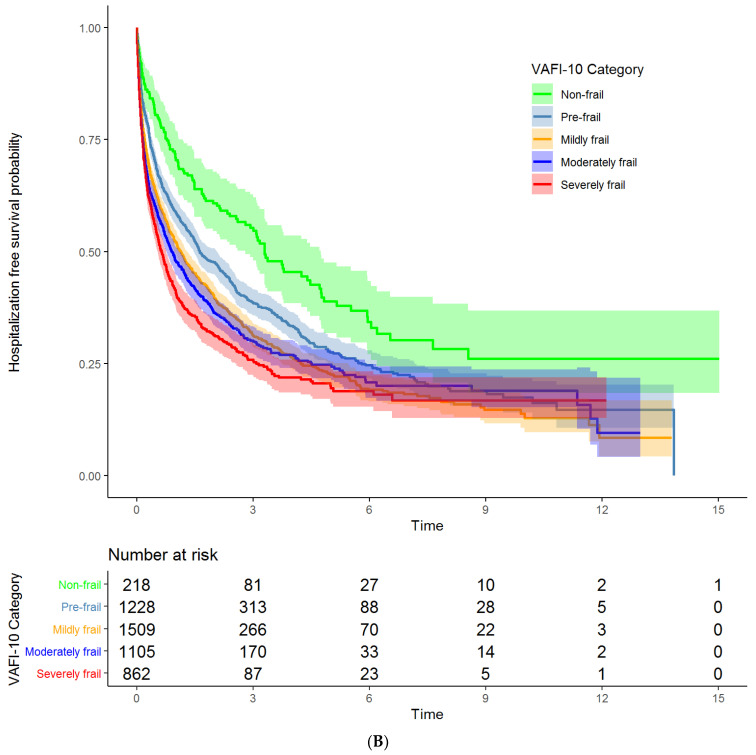
Kaplan–Meier curves demonstrating time (in years) to death (**A**) and time (in years) to hospitalization (**B**) by VA-FI-10 severity. Log-rank test *p*-value < 0.001 for both analyses.

Median follow-up was 2.40 years (IQR = 1.14, 4.14 years), and 3614 veterans (73.4%) died during the study period. Survival decreased across increasing VA-FI-10 severity (log-rank test *p* < 0.001, Figure 3A). Veterans classified as non-frail demonstrated the longest survival (median survival = 5.46 years, 95% confidence interval (CI) = 3.99 to 6.60 years), whereas veterans classified as severely frail demonstrated the shortest survival (median survival = 1.52 years, 95% CI = 1.39 to 1.74 years). The 1- and 5-year survival probability by VA-FI-10 severity were as follows: non-frail = 92.6% (95% CI = 89.2% to 96.2%) and 52.1% (45.2% to 60.0%); pre-frail = 85.8% (83.9% to 87.8%) and 40.8% (37.9% to 44.0%); mildly frail = 79.8% (77.8% to 81.8%) and 30.7% (28.2% to 33.4%); moderately frail = 73.7% (71.1% to 76.3%) and 20.4% (17.9% to 23.3%); and severely frail = 63.6% (60.4% to 66.9%) and 14.6% (12.1% to 17.7%).

In univariable Cox regression, mortality increased with increasing VA-FI-10 severity: compared to non-frail veterans, pre-frail veterans had a 1.33 times higher hazard of death (95% CI = 1.10 to 1.61), and this hazard ratio increased with VA-FI-10 severity up to 3.11 for severely frail veterans (95% CI = 2.56 to 3.77; Table 3). This “dose-response” relationship between VA-FI-10 and mortality was slightly attenuated but maintained in multivariable Cox regression adjusting for age, sociodemographic variables, year of treatment initiation, and measures of disease risk (Table 3; effect estimates for all covariates are presented in Table S2).

Similarly, time from treatment initiation to first unplanned hospitalization decreased across increasing frailty severity category (log-rank test *p* < 0.001, Figure 3B). In univariable Cox regression, the hazard of hospitalization increased with increasing VA-FI-10 severity: compared to non-frail veterans, pre-frail veterans had a 1.42 times higher hazard of hospitalization (95% CI = 1.18 to 1.73), and this hazard ratio increased with VA-FI-10 severity up to 2.11 (95% CI = 1.73 to 2.58; Table 3). This “dose–response” relationship between VA-FI-10 and time to hospitalization was slightly attenuated but maintained in multivariable Cox regression adjusting for age, sociodemographic variables, year of treatment initiation, and measures of disease risk (Table 3; effect estimates for all covariates presented in Table 2). Results were similar in complete case analyses as well as sensitivity analyses restricted to years 2012–2017 (Tables S3 and S4).

### 3.3. Impact on VA-FI-10 of Adding CMS Data to Capture External Deficits and of Varying Assessment Periods

The addition of CMS data (capturing external health deficits) to VA CDW data shifted the distribution of the VA-FI-10 toward higher frailty (median VA-FI-10 without CMS data = 0.23, IQR = 0.16 to 0.33; median VA-FI-10 with CMS data = 0.26, IQR = 0.19 to 0.35; Figure 2A, blue vs. red histogram). Using VA CDW data alone, 2919 veterans (58.9%) were classified as frail (VA-FI-10 > 0.2), compared to 3477 veterans (70.6%) when using CMS data in addition to VA CDW data. With the addition of CMS data, the mean increase in VA-FI-10 for each veteran was 0.04 (95% CI = 0.03 to 0.04, *p*-value < 0.001), with 24.2% of veterans reclassified to a higher VA-FI-10 severity category (e.g., from pre-frail to mildly frail), and 12.4% increasing by a VA-FI-10 value of 0.1 or greater (e.g., from 0.10 to 0.25). Adding CMS data to VA CDW data did not substantially change the association of VA-FI-10 with mortality (Table S5), but did result in lower HRs for unplanned VA hospitalizations.

Compared to a 1-year assessment period to measure health deficits prior to the index date, using a 3-year assessment period shifted its distribution towards higher frailty levels (median VA-FI-10 with 1-year assessment period = 0.23, IQR = 0.16 to 0.29; median VA-FI-10 with 3-year assessment period = 0.26, IQR = 0.19 to 0.35; Figure 2B, blue vs. red histogram). Using a 1-year assessment period, 2686 veterans (54.5%) were classified as frail (VA-FI-10 > 0.2), compared to 3477 veterans (70.6%) when using a 3-year assessment period. When increasing from a 1-year to a 3-year assessment period, the mean increase in VA-FI-10 for each veteran was 0.05 (95% CI = 0.05 to 0.05, *p*-value < 0.001), with 42.5% of veterans reclassified to a higher VA-FI-10 severity category, and 13.0% increasing by a VA-FI-10 value of 0.1 or greater. Despite this, using a 1- vs. 3-year assessment period did not substantially change the association of VA-FI-10 with mortality or hospitalizations (Table S6).

## 4. Discussion

When estimated using the VA-FI-10, frailty is prevalent in older transplant-ineligible US veterans newly treated for MM in VA. The VA-FI-10 strongly predicted survival and hospitalizations, independently of age, sociodemographic variables, and measures of disease risk. The inclusion of CMS data with VA CDW data and the use of a 1-year assessment window instead of a 3-year assessment window reclassified frailty severity for a substantial portion of veterans. Despite this, the VA-FI-10’s associations with outcomes were largely maintained across data sources and assessment periods.

The strong associations found between the VA-FI-10 and mortality reinforce the findings of others investigating FIs and eFIs in older populations with MM and other cancers [43,44,45,46,47]. In particular, Patel et al. studied in veterans aged 65 and older with MM the original ICD-9-based VA-FI through 2014, finding it to be independently associated with mortality [43]. We show that this association between frailty and mortality is maintained through the ICD-10 years of 2015–2017, and further expand the predictive validity of the VA-FI-10 to unplanned hospitalizations. Moreover, we show that there was little difference in the associations between the VA-FI-10 and mortality when adding CMS data to VA CDW data and changing from a 1-year to 3-year assessment period. The stronger association between hospitalizations and the VA-FI-10 measured from VA CDW data alone likely stems from our measurement of hospitalizations within VA only (i.e., veterans with a larger proportion of health deficits managed inside VA will be more likely to utilize VA hospitals compared to veterans with more health deficits managed outside VA). Altogether, our findings show that in older veterans with MM, the VA-FI-10 is a significant, independent predictor of mortality and care utilization regardless of the data sources and assessment periods used for its measurement. Accordingly, it can be used as an exposure and covariate in population-based studies, even by researchers who may not have access to CMS data to capture health deficits managed outside VA.

Compared to the VA-FI-10, frailty scores developed specifically for myeloma patients, such as the International Myeloma Working Group Frailty Score (IMWG-FS) and the revised Myeloma Comorbidity Index (R-MCI), are derived from geriatric measures collected in person from patients (e.g., patient-reported activities of daily living (ADLs)) [5,48]. These myeloma frailty scores are more widely studied and validated in risk stratification and prediction. Moreover, the VA-FI-10’s use of administrative diagnostic and procedure codes to measure important deficits contributing to frailty (e.g., functional dependency) can lack sensitivity, given the low rates of screening and billing for these geriatric health deficits compared to disease-based codes (e.g., cardiovascular procedures) that are better reimbursed in clinical practice [49,50].

However, the VA-FI-10 is based on one of the most widely studied models of frailty in aging research in deficit-accumulation [30]. The advantages of a deficit-accumulation frailty index are that it covers not only function and comorbidity but also cognition, sensory, and other geriatric domains—more comprehensively assessing for health deficits across multiple systems that additively reflect lower physiologic reserves. Rather than chronologic age—which is included in the IMWG-FS and R-MCI and may contribute to inaccurately classifying fit patients aged ≥ 75 years as frail—an assessment of physiologic age better distinguishes frailty severity even among the oldest of the old [8,51,52]. Future work should compare the classification and predictive performance of the cumulative deficit model of frailty with myeloma frailty scores, and ultimately investigate which scores best optimize treatment decisions.

Finally, the main advantage of the electronic VA-FI-10 is that it can estimate frailty from routinely collected data without requiring in-person assessment of geriatric measures, the implementation of which are limited by time and staff in busy oncology clinics [11,53]. The VA-FI-10 thus has the potential to not only more comprehensively assess geriatric domains compared to myeloma frailty scores, but to do so more rapidly with little user burden. With a large national healthcare system that provides a high degree of care continuity, such as the VA, the VA-FI-10’s electronic measurement of frailty can be used retrospectively to evaluate differences in outcomes among older patients who did not have dedicated geriatric or frailty assessments [11,53]. Moreover, this automated and “passive” assessment of frailty holds the promise of being used to prospectively screen for frailty without the need of a geriatrician in the clinic or additional assessments by the busy oncologist [18,54].

Our findings also highlight the urgent need of expanding frailty assessment in clinical practice, and the potential role of the VA-FI-10 in doing so. Nearly 70.6% of veterans with MM were classified as frail in contrast to 39–44% of veterans in cohorts representing a more general veteran population [21]. In 2017, veterans with MM had over double the rates of anemia, chronic kidney disease (CKD), chronic pain, and weight loss, likely reflecting the comorbidities arising from MM. Coexisting with these conditions was a high burden of cardiovascular disease, diabetes, functional limitations, and cognitive and sensory impairments—reflecting the aging-related conditions that clinicians must consider alongside an older veteran’s MM. The ability of the VA-FI-10 to capture these nononcologic chronic conditions contrasts with other eFIs used in other data sources in which there is low capture of chronic conditions [16]. Screening for functional and cognitive impairment is of particular importance in MM and other blood cancers, which entail increasingly more complex treatment regimens that require mobilization to frequent clinic appointments and increased use of novel oral agents necessitating reliable self-administration at home [55]. The VA-FI-10’s ability to reflect both MM-associated and aging-related health deficits allows for estimation of not only summative frailty but also its individual contributors, revealing deficits that may be improved, or worsened, by MM and its treatment.

However, our demonstration of shifts in the distribution of VA-FI-10 that result from varying data sources and assessment periods suggests a need for caution in the use of its cutoffs for classifying individuals into categories of frailty severity. A veteran can shift from pre-frail to frail or from mild frailty to more severe frailty simply by changes in the way the VA-FI-10 is measured, not by changes in their actual frailty. On average, the change for each veteran was small (~0.04 with addition of CMS data and ~0.05 with increasing from a 1-year to 3-year assessment period), below what is considered a large clinically meaningful change for an FI in community-dwelling older adults [56]. These small average changes, all in the same direction toward more severe frailty, in part explain why—at a population level—the associations between frailty and outcomes were strong regardless of data sources and length of assessment period.

At an individual level, however, these changes in frailty classification have implications for any future use of the VA-FI-10 in clinical practice. The choice of initial intensity of MM therapy, varying in number and doses of agents, rests largely on the measured frailty of the patient [4]. Given the variety of measures and cutoffs with which to classify frailty in older patients with MM and low agreement among some measures [51,52,57], deeming one “frail” should not predetermine a lower intensity therapy (e.g., doublet therapy instead of triplet therapy) and vice versa. Instead, understanding how frailty can inform the management of an individual first requires an awareness of the criteria and measurement properties of the particular frailty measure used. Our analyses additionally reveal that this maxim holds true for an eFI such as the VA-FI-10, which requires knowledge of whether the standard method of measurement (CMS + VA CDW data with a 3-year assessment period) or some variation is being used. Failing to consider the method of measurement of the VA-FI-10 and its influence on frailty classification may risk overtreatment in frail veterans estimated to be fit by the VA-FI-10, as well as undertreatment of fit veterans overestimated to be frail [58].

For example, Patel et al. used the original ICD-9-based VA-FI with a 1-year assessment period and without CMS data in their study of the VA-FI in older veterans with MM [43]. Along with the increase in frailty with CMS data, we showed that a 3-year assessment period compared to a 1-year period reclassifies over 40% of veterans to a more severe frailty category due to the capture of additional health deficits coded prior to the year preceding myeloma treatment. The degree with which this additional capture of health deficits increases the sensitivity of the VA-FI-10 for detecting true frailty must be balanced with the degree to which it decreases its specificity. This balance can only be ascertained in future work comparing the electronic VA-FI-10 with an in-person frailty index, as has been done by Kim et al. for the US Medicare-based eFI [59] and Clegg et al. for the UK’s NHS eFI [60].

There are limitations to our study. First, our inclusion criteria require that veterans with MM survive until treatment, potentially excluding those who die before receiving treatment. However, most patients with newly diagnosed MM are indicated for immediate treatment, and only using MM diagnostic codes for selection is nonspecific for identifying patients with true MM [61]. Moreover, the risk of immortal time bias in requiring a second treatment code after the index date is low given that the median time between the first and second treatment codes is only 11 days (IQR = 4–29 days). Second, selecting for veterans treated for their MM within VA limits the application of our results in veterans treated outside VA. Excluding veterans whose MM was managed outside VA decreased the missingness of our covariates, since the CMS data are more limited than the VA data with regard to labs and sociodemographic variables. Moreover, excluding these veterans minimizes misclassification of treatment and the date of treatment initiation (index date). Third, our results are potentially subject to residual confounding by the unmeasured MM cytogenetics. Data on cytogenetics are often in unstructured data and more difficult to access than the other laboratory covariates used in our study. Our results are also subject to residual confounding by treatment intensity, i.e., whether a patient received a 2-drug, 3-drug, or 4-drug regimen for their induction treatment. Although we currently can measure the date and type of individual treatments prescribed, we cannot confirm with high reliability the initial intensity of treatment. Accurate treatment classification will allow for future analyses investigating treatment as a mediator and the VA-FI-10 as a modifier of the effect that initial treatment intensity has on outcomes. Finally, we did not measure external hospitalizations, and time-to-hospitalization was likely affected by the competing risk of death.

## 5. Conclusions

Our findings support the use of the VA-FI-10, even when measured without CMS data and with varying assessment periods, in future research aiming to investigate outcomes in frail veterans treated with contemporary MM therapies that are studied predominantly in younger and fitter trial populations. The VA-FI-10 also holds promise as a tool to scale frailty assessment in VA oncology practice, but the level of frailty severity assigned to an individual can be influenced by the way in which the VA-FI-10 is measured. This measurement variability calls for caution before using an eFI like the VA-FI-10 to inform selection of MM treatment intensity. Future research should compare the VA-FI-10 to an in-person frailty index, create cross-walks between a VA-FI-10 using VA data only and a VA-FI-10 using VA + CMS data, and study whether varying data sources and assessment periods affects individual prognosis. In the meantime, future clinical use of the VA-FI-10 to estimate frailty in individual veterans with MM should be paired with a validated in-person frailty measure, such a gait speed, or with a geriatric assessment (similar to current practice with the NHS eFI [18]), depending on clinic resources. Such an approach would offer a practical alternative to delivering a comprehensive geriatric assessment for every older veteran with MM—an ideal that is difficult to scale—while still advancing frailty assessment beyond the status quo.

## Figures and Tables

**Table 1:** Baseline characteristics of 4924 veterans with MM according to VA-FI-10 severity.

Characteristic	Overall	Non-Frail (VA-FI ≤ 0.1)	Pre-Frail (VA-FI > 0.1–0.2)	Mildly Frail (VA-FI > 0.2–0.3)	Moderately Frail (VA-FI > 0.3–0.4)	Severely Frail (VA-FI > 0.4)
*n*	4924	219	1228	1510	1105	862
Age at Diagnosis (median (IQR))	75.1 (69.9, 80.8)	72.6 (68.7, 77.3)	73.1 (68.7, 78.5)	75.0(69.9, 80.6)	76.5 (70.5, 81.7)	77.4 (72.0, 82.8)
Gender = M (%)	4857 (98.6)	217 (99.1)	1220 (99.3)	1486 (98.4)	1088 (98.5)	846 (98.1)
Race (%)						
White	3273 (66.5)	144 (65.8)	818 (66.6)	983 (65.1)	726 (65.7)	602 (69.8)
Black	1117 (22.7)	42 (19.2)	283 (23.0)	369 (24.4)	252 (22.8)	171 (19.8)
Other	64 (1.3)	5 (2.3)	19 (1.5)	15 (1.0)	14 (1.3)	11 (1.3)
Missing	470 (9.5)	28 (12.8)	108 (8.8)	143 (9.5)	113 (10.2)	78 (9.0)
Income in US dollars (median (IQR))	27,519 (16,368, 38,647)	32,491 (19,892, 46,254)	29,078 (17,828, 42,646)	26,461 (16,000, 37,716)	26,507 (16,235, 37,678)	26,720 (15,506, 35,916)
ISS Stage (%)						
1	495 (10.1)	37 (16.9)	171 (13.9)	154 (10.2)	87 (7.9)	46 (5.3)
2	924 (18.8)	40 (18.3)	262 (21.3)	286 (18.9)	204 (18.5)	132 (15.3)
3	626 (12.7)	22 (10.0)	127 (10.3)	210 (13.9)	156 (14.1)	111 (12.9)
Missing	2879 (58.5)	120 (54.8)	668 (54.4)	860 (57.0)	658 (59.5)	573 (66.5)
Calcium ≥ 11 mg/dL (%)	151 (3.1)	4 (1.8)	40 (3.3)	46 (3.0)	39 (3.5)	22 (2.6)
Missing	422 (8.6)	27 (12.3)	108 (8.8)	131 (8.7)	83 (7.5)	73 (8.5)
Creatinine > 2 mg/dL (%)	997 (20.2)	11 (5.0)	160 (13.0)	330 (21.9)	259 (23.4)	237 (27.5)
Missing	347 (7.0)	23 (10.5)	92 (7.5)	105 (7.0)	62 (5.6)	65 (7.5)
Hemoglobin < 10 g/dL (%)	1727 (35.1)	41 (18.7)	362 (29.5)	532 (35.2)	425 (38.5)	367 (42.6)
Missing	347 (7.0)	27 (12.3)	92 (7.5)	100 (6.6)	71 (6.4)	57 (6.6)
Platelet < 150,000/microL (%)	1116 (22.7)	38 (17.4)	262 (21.3)	328 (21.7)	274 (24.8)	214 (24.8)
Missing	626 (12.7)	37 (16.9)	154 (12.5)	191 (12.6)	142 (12.9)	102 (11.8)
Novel Therapy at Induction (%)	4231 (85.9)	198 (90.4)	1056 (86.0)	1308 (86.6)	937 (84.8)	732 (84.9)
Thalidomide (%)	1089 (22.1)	48 (21.9)	252 (20.5)	355 (23.5)	250 (22.6)	184 (21.3)
Lenalidomide (%)	1870 (38.0)	108 (49.3)	526 (42.8)	574 (38.0)	383 (34.7)	279 (32.4)
Bortezomib (%)	1990 (40.4)	77 (35.2)	497 (40.5)	618 (40.9)	434 (39.3)	364 (42.2)
Thalidomide and Bortezomib (%)	70 (1.4)	1 (0.2)	28 (1.7)	25 (1.7)	10 (1.2)	6 (1.1)
Lenalidomide and Bortezomib (%)	633 (12.9)	44 (10.7)	211 (13.1)	211 (14.0)	-	-

**Table 2:** Prevalence of VA-FI-10 health deficits for 4924 veterans with MM overall and by VA-FI severity.

Health Deficit	Overall	Non-Frail (VA-FI ≤ 0.1)	Pre-Frail (VA-FI > 0.1–0.2)	Mildly Frail (VA-FI > 0.2–0.3)	Moderately Frail (VA-FI > 0.3–0.4)	Severely Frail (VA-FI > 0.4)
*n* (%)	4924 (100)	219 (4.4)	1228 (24.9)	1510 (30.7)	1105 (22.4)	862 (17.5)
Morbidity						
Atrial Fibrillation	928 (18.8)	2 (0.9)	70 (5.7)	211 (14.0)	289 (26.2)	356 (41.3)
Anemia	3629 (73.7)	55 (25.1)	690 (56.2)	1147 (76.0)	946 (85.6)	791 (91.8)
Coronary Artery Disease	2071 (42.1)	15 (6.8)	227 (18.5)	560 (37.1)	632 (57.2)	637 (73.9)
Cancer	4813 (97.7)	199 (90.9)	1185 (96.5)	1486 (98.4)	1088 (98.5)	855 (99.2)
Cerebral Vascular Disease	996 (20.2)	1 (0.5)	61 (5.0)	216 (14.3)	304 (27.5)	414 (48.0)
Chronic Kidney Disease	2043 (41.5)	7 (3.2)	272 (22.1)	587 (38.9)	583 (52.8)	594 (68.9)
Diabetes	2019 (41.0)	15 (6.8)	293 (23.9)	588 (38.9)	559 (50.6)	564 (65.4)
Heart Failure	1144 (23.2)	2 (0.9)	47 (3.8)	236 (15.6)	377 (34.1)	482 (55.9)
Hypertension	4375 (88.9)	116 (53.0)	1002 (81.6)	1357 (89.9)	1051 (95.1)	849 (98.5)
Liver Disease	540 (11.0)	4 (1.8)	59 (4.8)	143 (9.5)	141 (12.8)	193 (22.4)
Lung Disease	1780 (36.1)	9 (4.1)	221 (18.0)	496 (32.8)	522 (47.2)	532 (61.7)
Thyroid Disease	739 (15.0)	4 (1.8)	87 (7.1)	226 (15.0)	182 (16.5)	240 (27.8)
Osteoporosis or Osteoporosis-Related Fracture	842 (17.1)	10 (4.6)	94 (7.7)	215 (14.2)	243 (22.0)	280 (32.5)
Incontinence	388 (7.9)	0 (0.0)	35 (2.9)	78 (5.2)	101 (9.1)	174 (20.2)
Function						
Arthritis	2754 (55.9)	36 (16.4)	462 (37.6)	851 (56.4)	743 (67.2)	662 (76.8)
Durable Medical Equipment	1102 (22.4)	6 (2.7)	104 (8.5)	277 (18.3)	315 (28.5)	400 (46.4)
Falls	550 (11.2)	3 (1.4)	25 (2.0)	115 (7.6)	150 (13.6)	257 (29.8)
Fatigue	1274 (25.9)	7 (3.2)	91 (7.4)	301 (19.9)	375 (33.9)	500 (58.0)
Gait Abnormality	1019 (20.7)	0 (0.0)	61 (5.0)	209 (13.8)	321 (29.0)	428 (49.7)
Muscular impairment/Debility	941 (19.1)	3 (1.4)	54 (4.4)	165 (10.9)	278 (25.2)	441 (51.2)
Parkinson’s Disease	151 (3.1)	0 (0.0)	14 (1.1)	31 (2.1)	37 (3.3)	69 (8.0)
Peripheral Vascular Disease/Claudication	1512 (30.7)	7 (3.2)	143 (11.6)	371 (24.6)	457 (41.4)	534 (61.9)
Cognition and Mood						
Dementia	685 (13.9)	1 (0.5)	47 (3.8)	130 (8.6)	208 (18.8)	299 (34.7)
Anxiety	622 (12.6)	3 (1.4)	68 (5.5)	150 (9.9)	163 (14.8)	238 (27.6)
Depression	1155 (23.5)	8 (3.7)	137 (11.2)	275 (18.2)	325 (29.4)	410 (47.6)
Sensory Loss						
Peripheral Neuropathy	582 (11.8)	2 (0.9)	25 (2.0)	115 (7.6)	181 (16.4)	259 (30.0)
Hearing Impairment	1693 (34.4)	23 (10.5)	282 (23.0)	491 (32.5)	459 (41.5)	438 (50.8)
Vision Impairment	1510 (30.7)	15 (6.8)	225 (18.3)	433 (28.7)	422 (38.2)	415 (48.1)
Other						
Chronic Pain	1416 (28.8)	10 (4.6)	166 (13.5)	389 (25.8)	386 (34.9)	465 (53.9)
Failure to Thrive	86 (1.7)	0 (0.0)	1 (0.1)	12 (0.8)	26 (2.4)	47 (5.5)
Weight Loss	598 (12.1)	2 (0.9)	64 (5.2)	148 (9.8)	186 (16.8)	198 (23.0)

**Table 3:** Univariable and multivariable Cox proportional hazards regression models estimating the association of VA-FI-10 with overall mortality and time to hospitalization among 4294 veterans with MM.

VA-FI-10 Severity	Mortality Unadjusted HR (95% CI)	Mortality Adjusted HR (95% CI)	Hospitalization Unadjusted HR (95% CI)	Hospitalization Adjusted HR (95% CI)
Non-frail	Reference	Reference	Reference	Reference
Pre-frail	1.33 (1.10 to 1.61)	1.25 (1.04 to 1.52)	1.42 (1.18 to 1.73)	1.32 (1.08 to 1.60)
Mildly frail	1.76 (1.46 to 2.13)	1.54 (1.27 to 1.86)	1.72 (1.42 to 2.08)	1.58 (1.31 to 1.92)
Moderately frail	2.35 (1.94 to 2.84)	1.95 (1.61 to 2.37)	1.82 (1.50 to 2.21)	1.69 (1.39 to 2.06)
Severely frail	3.11 (2.56 to 3.77)	2.50 (2.05 to 3.04)	2.11 (1.73 to 2.58)	1.93 (1.58 to 2.36)

All adjusted analyses were on imputed data. Models were adjusted for all covariates, including age at MM diagnosis, gender, race, income, year of treatment initiation, ISS stage, calcium greater than or equal to 11 mg/dL, creatinine greater than 2 mg/dL, hemoglobin less than 10 g/dL, and platelets less than 150,000/microL. HR = hazard ratio; CI = confidence interval.

## Data Availability

The data underlying this article were accessed from the VA Corporate Data Warehouse. The derived data generated in this research may be shared on reasonable request to the corresponding author as permitted by VA policy. We have also uploaded code to compute the VA Frailty Index here: https://github.com/bostoninformatics/va_frailty_index, (accessed on 10 January 2020).

## Supplementary Materials

The following are available online at https://www.mdpi.com/article/10.3390/cancers13123053/s1, Table S1: Complete case analysis involving multivariable Cox proportional hazards regression models estimating the association of VA-FI-10 with overall mortality and hospitalizations, Table S2: Multivariable Cox proportional hazards regression models estimating the association of VA-FI-10 with overall mortality and hospitalizations, using data only from VA Corporate Data Warehouse (CDW) to measure health deficits in VA-FI-10, Table S3: Complete case analysis involving multivariable Cox proportional hazards regression models estimating the association of VA-FI-10 with overall mortality and hospitalizations, Table S4: Sensitivity analysis in years 2012–2017, repeating multivariable Cox proportional hazards regression models for mortality and hospitalizations to assess whether associations with VA-FI-10 hold in the later time period of the study during which a higher proportion of patients were receiving modern-era treatments, Table S5: Multivariable Cox proportional hazards regression models estimating the association of VA-FI-10 with overall mortality and hospitalizations, using data only from VA Corporate Data Warehouse (CDW) to measure health deficits in VA-FI-10, Table S6: Multivariable Cox proportional hazards regression models estimating the association of VA-FI-10 with overall mortality and hospitalizations, using data from VA Corporate Data Warehouse (CDW) and Centers for Medicare and Medicaid Services (CMS) and 1-year assessment period to measure health deficits in VA-FI-10.

## Appendix A

Supplemental Methods: Under “Data Source and Population” subheading: Excluding veterans who did not routinely use VA or whose myeloma was managed outside VA decreased the missingness of our covariates, since the CMS data are more limited than the VA data with regard to lab and sociodemographic variables. Increasing missingness of key covariates would have increased the risk of confounding given less robust adjustment. Moreover, excluding these veterans minimized the misclassification of the treatment and index date—when the veteran first initiated treatment for their myeloma within VA. Under the “Measurement of Frailty and Covariates” subheading: Deficits in the VA-FI-10 were measured using 6422 unique International Classification of Diseases (ICD) diagnostic codes, Current Procedural Terminology (CPT) codes, and Healthcare Common Procedure Coding Systems (HCPCS) codes. The VA-FI-10 includes both ICD-9 and ICD-10 codes for measurement of frailty before and after the transition to ICD-10, which occurred in the US on October 1, 2015. See “Data Availability Statement” for code to compute the VA-FI-10.
