# Supplementary material for: Contemporary Analysis of Electronic Frailty Measurement in Older Adults with Multiple Myeloma Treated in the National US Veterans Affairs Healthcare System
Source: Cancers (Basel). 2021 Jun 18;13(12):3053. doi: 10.3390/cancers13123053 (PMC8233717; doi:10.3390/cancers13123053)
Supplement: Supplementary file 1 [file cancers-13-03053-s001.zip › cancers-1237955-supplementary.pdf]

Supplementary Material: Contemporary Analysis of Electronic Frailty Measurement in Older Adults with Multiple Myeloma Treated in the National US Veterans Affairs Healthcare System

Clark DuMontier, Nathanael R. Fillmore, Cenk Yildirim, David Cheng, Jennifer La, Ariela R. Orkaby, Brian Charest, Diana Cirstea, Sarvari Yellapragada, John Michael Gaziano, Nhan Do, Mary T. Brophy, Dae H. Kim, Nikhil C. Munshi and Jane A. Driver

Table S1. Prevalence of VA-FI-10 health deficits for 4,924 Veterans with MM by cohort year.

Health Deficit	2012	2013	2014	2015	2016	2017
n (%)	284 (100)	358 (100)	439 (100)	437 (100)	487 (100)	499 (100)
Morbidity						
Atrial fibrillation	49 (17.3)	66 (18.4)	77 (17.5)	84 (19.2)	94 (19.3)	91 (18.2)
Anemia	195 (68.7)	249 (69.6)	313 (71.3)	308 (70.5)	353 (72.5)	332 (66.5)
Coronary Artery Disease	102 (35.9)	137 (38.3)	178 (40.5)	159 (36.4)	187 (38.4)	186 (37.3)
Cancer	276 (97.2)	348 (97.2)	429 (97.7)	424 (97.0)	476 (97.7)	485 (97.2)
Cerebral Vascular Disease	52 (18.3)	63 (17.6)	81 (18.5)	73 (16.7)	78 (16.0)	99 (19.8)
Chronic Kidney Disease	115 (40.5)	167 (46.6)	195 (44.4)	177 (40.5)	215 (44.1)	216 (43.3)
Diabetes	127 (44.7)	149 (41.6)	200 (45.6)	177 (40.5)	216 (44.4)	234 (46.9)
Heart Failure	65 (22.9)	87 (24.3)	99 (22.6)	89 (20.4)	98 (20.1)	102 (20.4)
Hypertension	247 (87.0)	323 (90.2)	394 (89.7)	377 (86.3)	422 (86.7)	444 (89.0)
Liver Disease	23 (8.1)	35 (9.8)	51 (11.6)	50 (11.4)	52 (10.7)	50 (10.0)
Lung Disease	92 (32.4)	115 (32.1)	156 (35.5)	151 (34.6)	175 (35.9)	180 (36.1)
Thyroid Disease	42 (14.8)	43 (12.0)	66 (15.0)	51 (11.7)	73 (15.0)	78 (15.6)
Osteoporosis or osteoporosis-related fracture	41 (14.4)	57 (15.9)	62 (14.1)	78 (17.8)	62 (12.7)	58 (11.6)
Incontinence	30 (10.6)	26 (7.3)	35 (8.0)	31 (7.1)	36 (7.4)	45 (9.0)
Function						
Arthritis	172 (60.6)	197 (55.0)	247 (56.3)	240 (54.9)	257 (52.8)	256 (51.3)
Durable Medical Equipment	59 (20.8)	82 (22.9)	96 (21.9)	100 (22.9)	143 (29.4)	175 (35.1)
Falls	27 (9.5)	35 (9.8)	52 (11.8)	46 (10.5)	40 (8.2)	58 (11.6)
Fatigue	49 (17.3)	67 (18.7)	123 (28.0)	108 (24.7)	122 (25.1)	153 (30.7)
Gait Abnormality	60 (21.1)	73 (20.4)	107 (24.4)	101 (23.1)	112 (23.0)	126 (25.3)
Muscular impairment/debility	67 (23.6)	90 (25.1)	105 (23.9)	91 (20.8)	82 (16.8)	96 (19.2)
Parkinson’s Disease	12 (4.2)	11 (3.1)	15 (3.4)	13 (3.0)	15 (3.1)	15 (3.0)
Peripheral Vascular Disease/Claudication	85 (29.9)	103 (28.8)	118 (26.9)	126 (28.8)	122 (25.1)	136 (27.3)
Cognition and Mood						
Dementia	42 (14.8)	40 (11.2)	63 (14.4)	50 (11.4)	71 (14.6)	85 (17.0)
Anxiety	29 (10.2)	48 (13.4)	72 (16.4)	60 (13.7)	88 (18.1)	82 (16.4)
Depression	71 (25.0)	94 (26.3)	139 (31.7)	107 (24.5)	139 (28.5)	156 (31.3)
Sensory Loss						
Peripheral Neuropathy	29 (10.2)	36 (10.1)	52 (11.8)	47 (10.8)	79 (16.2)	120 (24.0)
Hearing Impairment	100 (35.2)	120 (33.5)	160 (36.4)	163 (37.3)	183 (37.6)	213 (42.7)
Vision Impairment	96 (33.8)	105 (29.3)	132 (30.1)	141 (32.3)	146 (30.0)	136 (27.3)
Other						
Chronic pain	70 (24.6)	100 (27.9)	122 (27.8)	146 (33.4)	157 (32.2)	146 (29.3)
Failure to Thrive	9 (3.2)	7 (2.0)	7 (1.6)	10 (2.3)	9 (1.8)	14 (2.8)
Weight loss	32 (11.3)	50 (14.0)	58 (13.2)	58 (13.3)	55 (11.3)	63 (12.6)

Table S2. Adjusted effect estimates for all variables included in multivariable Cox proportional hazards regression models estimating the association of VA-FI-10 with overall mortality and time to hospitalization.

Variable	Mortality HR	95% CI Lower Bound	95% CI Upper Bound	Hospitalization HR	95% CI Lower Bound	95% CI Upper Bound
Pre-frail	1.254	1.035	1.520	1.315	1.084	1.597
Mildly frail	1.539	1.273	1.861	1.584	1.307	1.919
Moderately frail	1.954	1.610	2.370	1.690	1.388	2.056
Severely frail	2.497	2.050	3.040	1.931	1.578	2.361
Age at Tx Initiation	1.030	1.025	1.037	0.986	0.980	0.991
Year of Tx Initiation	0.968	0.959	0.976	1.021	1.012	1.030
Black Race	0.890	0.821	0.965	1.123	1.036	1.219
Other Race	1.105	0.807	1.513	1.038	0.763	1.413
Gender = M	1.234	0.916	1.660	0.920	0.676	1.251
Income ≥ $15,000	0.839	0.770	0.913	0.828	0.757	0.904
Income Missing	0.909	0.801	1.033	0.555	0.477	0.647
ISS Stage 2	1.172	1.061	1.294	1.102	0.989	1.229
ISS Stage 3	1.223	1.068	1.399	1.102	0.933	1.301
Creatinine > 2	1.201	1.094	1.319	1.202	1.058	1.365
Calcium ≥ 11	1.317	1.087	1.594	1.420	1.175	1.718
Hemoglobin < 10	1.324	1.221	1.436	1.384	1.279	1.498
Platelet < 150	1.188	1.099	1.285	1.029	0.949	1.117

Table S3. Complete case analysis involving multivariable Cox proportional hazards regression models estimating the association of VA-FI-10 with overall mortality and hospitalizations.

VA-FI-10 Severity	Mortality HR (95% CI)	Hospitalization HR (95% CI)
Nonfrail	Reference	Reference
Pre-frail	1.26 (0.89 to 1.78)	1.24 (0.92 to 1.67)
Mildly frail	1.62 (1.15 to 2.27)	1.53 (1.14 to 2.05)
Moderately frail	2.12 (1.50 to 3.00)	1.85 (1.37 to 2.50)
Severely frail	2.44 (1.71 to 3.49)	1.99 (1.45 to 2.72)

All adjusted analyses were on imputed data. Models were adjusted for all covariates, including age at MM diagnosis, gender, race, income, year of treatment initiation, ISS stage, calcium greater than or equal to 11 mg/dL, creatinine greater than 2 mg/dL, hemoglobin less than 10 g/dL, and platelets less than 150,000/μL. HR = Hazard ratio, CI = Confidence interval.

Table S4. Sensitivity analysis in years 2012-2017, repeating multivariable Cox proportional hazards regression models for mortality and hospitalizations to assess whether associations with VA-FI-10 hold in the later time period of the study during which a higher proportion of patients were receiving modern-era treatments.

Variable	Mortality HR	95% CI Lower Bound	95% CI Upper Bound	Hospitalization HR	95% CI Lower Bound	95% CI Upper Bound
Pre-frail	1.413	1.007	1.984	1.254	0.961	1.636
Mildly frail	1.732	1.239	2.421	1.645	1.265	2.140
Moderately frail	2.340	1.670	3.277	1.944	1.486	2.547
Severely frail	2.889	2.052	4.067	2.344	1.782	3.080

All adjusted analyses were on imputed data. Models were adjusted for all covariates, including age at MM diagnosis, gender, race, income, year of treatment initiation, ISS stage, calcium greater than or equal to 11 mg/dL, creatinine greater than 2 mg/dL, hemoglobin less than 10 g/dL, and platelets less than 150,000/microL. HR = Hazard ratio, CI = Confidence interval.

Table S5. Multivariable Cox proportional hazards regression models estimating the association of VA-FI-10 with overall mortality and hospitalizations, using data only from VA Corporate Data Warehouse (CDW) to measure health deficits in VA-FI-10.

VA-FI-10 Severity	Mortality HR (95% CI)	Hospitalization HR (95% CI)
Nonfrail	Reference	Reference
Pre-frail	1.20 (1.05 to 1.38)	1.65 (1.40 to 1.94)
Mildly frail	1.55 (1.35 to 1.77)	2.32 (1.97 to 2.74)
Moderately frail	2.20 (1.90 to 2.55)	3.21 (2.70 to 3.81)
Severely frail	2.66 (2.26 to 3.12)	3.96 (3.29 to 4.76)

All adjusted analyses were on imputed data. Models were adjusted for all covariates, including age at MM diagnosis, gender, race, income, year of treatment initiation, ISS stage, calcium greater than or equal to 11 mg/dL, creatinine greater than 2 mg/dL, hemoglobin less than 10 g/dL, and platelets less than 150,000/μL. HR = Hazard ratio, CI = Confidence interval.

Table S6. Multivariable Cox proportional hazards regression models estimating the association of VA-FI-10 with overall mortality and hospitalizations, using data from VA Corporate Data Warehouse (CDW) and Centers for Medicare and Medicaid Services (CMS) and 1-year assessment period to measure health deficits in VA-FI-10.

VA-FI-10 Severity	Mortality HR (95% CI)	Hospitalization HR (95% CI)
Nonfrail	Reference	Reference
Pre-frail	1.24 (1.09 to 1.4)	1.33 (1.76 to 1.51)
Mildly frail	1.49 (1.31 to 1.69)	1.54 (1.35 to 1.76)
Moderately frail	2.13 (1.85 to 2.45)	1.85 (1.60 to 2.15)
Severely frail	2.75 (2.34 to 3.23)	2.11 (1.78 to 2.50)

All adjusted analyses were on imputed data. Models were adjusted for all covariates, including age at MM diagnosis, gender, race, income, year of treatment initiation, ISS stage, calcium greater than or equal to 11 mg/dL, creatinine greater than 2 mg/dL, hemoglobin less than 10 g/dL, and platelets less than 150,000/μL. HR = Hazard ratio, CI = Confidence interval.

Publisher's Note: MDPI stays neutral with regard to jurisdictional claims in published maps and institutional affiliations.

© 2021 by the authors. Licensee MDPI, Basel, Switzerland. This article is an open access article distributed under the terms and conditions of the Creative Commons Attribution (CC BY) license (http://creativecommons.org/licenses/by/4.0/).
